# Soluble urokinase receptor is a kidney-specific vasoconstrictor

**DOI:** 10.1016/j.ebiom.2025.106012

**Published:** 2025-11-03

**Authors:** Sebastian Buhl Rasmussen, Rasmus Bo Lindhardt, Georgina Gyarmati, Kirsten Madsen, Claus Bistrup, Lars Lund, Sisse Rye Ostrowski, Changli Wei, Janos Peti-Peterdi, Per Svenningsen, Jochen Reiser, Hanne Berg Ravn

**Affiliations:** a Department of Anaesthesiology and Intensive Care, Odense University Hospital, Odense, 5000, Denmark; b Department of Clinical Research, Faculty of Health Sciences, University of Southern Denmark, Odense, 5230, Denmark; c Department of Physiology and Neuroscience and Department of Medicine, Zilkha Neurogenetic Institute, University of Southern California, Los Angeles, CA, 90033, USA; d Department of Pathology, Odense University Hospital, Odense, 5000, Denmark; e Department of Molecular Medicine - Cardiovascular and Renal Research, University of Southern Denmark, Odense, 5230, Denmark; f Department of Nephrology, Odense University Hospital, Odense, 5000, Denmark; g Department of Urology, Odense University Hospital, Odense, 5000, Denmark; h Department of Clinical Immunology, Copenhagen University Hospital – Rigshospitalet, Copenhagen, 2100, Denmark; i Department of Clinical Medicine, Faculty of Health and Medical Sciences, University of Copenhagen, Copenhagen, 2200, Denmark; j Department of Internal Medicine, University of Texas Medical Branch, Galveston, TX, 77555, USA

**Keywords:** suPAR, Acute kidney injury, Vasoconstriction, Renal blood flow, Calcium signalling

## Abstract

**Background:**

Soluble urokinase plasminogen activator receptor (suPAR) is an innate immune system-derived risk factor for acute and chronic kidney diseases. While suPAR effects on kidney epithelial cells have been reported, its impact on renal vasculature remains unknown.

**Methods:**

We investigated how suPAR affects renal blood flow and glomerular dynamics using a translational approach integrating clinical observations from a propensity-score-matched cardiac surgery cohort, *ex vivo* porcine kidney perfusion, and intravital multiphoton imaging in mice.

**Findings:**

In the matched clinical cohort, we found a significant inverse correlation between suPAR levels and baseline kidney function, with mean eGFR values 14.5 mL/min/1.73 m^2^ lower in the high suPAR group (≥4 ng/mL) compared to the low suPAR group (<4 ng/mL). Patients with high suPAR levels had significantly higher AKI occurrence (56% vs 33%; relative risk 1.71, 95% CI 1.37–2.12). In experimental models, suPAR caused immediate reduction in renal blood flow and triggered robust calcium responses in renal contractile cells, particularly the extraglomerular mesangium. These effects were absent in brain vasculature and were antagonised by an anti-uPAR antibody.

**Interpretation:**

Unlike many immune mediators, suPAR causes predominantly kidney-specific vasoconstriction, establishing a new class of innate-immune vasoconstrictors with direct implications for causing acute kidney injury in high-risk patients.

**Funding:**

Supported by the 10.13039/100000002National Institutes of Health, 10.13039/501100006356University of Southern Denmark, Region of Southern Denmark PhD Fund, OUH-RH Joint Research Fund, Danish Kidney Association’s Research Fund, Odense University Hospital Research Fund, Goldsmith A. L. Rasmussen Memorial Fund, Dept. of Anaesthesiology-Intensive Care, OUH Research Fund and the MeCiSu Frontline Centre.

**Research in context**

**Evidence before this study**

Soluble urokinase plasminogen activator receptor (suPAR) is consistently associated with increased acute kidney injury risk across clinical settings. Previous studies established that suPAR sensitises kidney proximal tubules through cellular bioenergetics modulation and mediates inflammation via chemoattractant upregulation. However, suPAR's effects on renal vasculature remain unknown.

**Added value of this study**

We used a translational approach combining propensity-score-matched clinical data, *ex vivo* porcine perfusion, and intravital multiphoton imaging in mice to demonstrate that suPAR directly causes kidney-specific vasoconstriction. We revealed that suPAR triggered robust calcium responses in renal contractile cells, leading to afferent arteriole vasoconstriction comparable to stimulation with moderate doses of angiotensin II. These effects were kidney-specific, absent in brain vasculature, and could be completely prevented by anti-uPAR antibody.

**Implications of all the available evidence**

Our findings establish suPAR as an innate-immune vasoconstrictor with kidney specificity, providing mechanistic insight into the association between elevated suPAR levels and acute kidney injury, and suggest new therapeutic targets for preventing kidney injury in high-risk patients with elevated suPAR levels.

## Introduction

Acute kidney injury (AKI) is a common and life-threatening complication across various clinical settings, including critical care, major surgery, and severe infections, with substantial implications for patient outcomes, including increased mortality and progression to chronic kidney disease (CKD).^1^ Despite advancements in supportive care, identifying diverse mechanisms leading to different AKI phenotypes and developing targeted preventive interventions remain key challenges in clinical practice.^2^

The risk of kidney injury is amplified in individuals with preexisting kidney dysfunction or heightened inflammatory responses.^3^ One key inflammatory biomarker is the soluble urokinase plasminogen activator receptor (suPAR), a soluble form of the glycosylphosphatidylinositol-anchored three-domain membrane protein urokinase-type plasminogen activator receptor (uPAR), expressed on various cells, including endothelial and immune-active cells.^4^ Elevated suPAR levels have consistently been associated with a higher frequency and severity of AKI across different clinical settings.^5^^,^^6^ While the association between suPAR and AKI is well-established, the mechanisms through which suPAR contributes to kidney injury are not fully understood. Evidence suggests suPAR sensitises kidney proximal tubules to injury by modulating cellular bioenergetics and increasing oxidative stress.^7^ Additionally, suPAR mediates kidney inflammation, exacerbating AKI through upregulation of chemoattractants and T-cell infiltration.^8^ However, while suPAR's effects on epithelial cells are increasingly understood, its potential effect on renal vasculature remains unexplored.

The relationship between suPAR and glomerular filtration rate (GFR) has been evaluated in several studies. Still, both biomarkers are influenced by comorbidities such as cardiovascular disease, diabetes, advanced age, and sex differences, making it challenging to tease out their contributions.^9^, ^10^, ^11^ The primary aim of this study was to investigate how suPAR interferes with renal pathophysiology, particularly its effects on renal blood flow and glomerular dynamics, using a translational approach. We employed propensity score matching to examine the relationship between suPAR levels and baseline kidney function in cardiac surgery patients, complemented by mechanistic studies using *ex vivo* porcine kidney perfusion and *in vivo* rodent models to investigate the underlying vascular mechanisms.

## Methods

### Human samples

To investigate the relationship between suPAR levels and baseline kidney function, plasma suPAR values were analysed from biobanked blood samples of 913 patients undergoing elective on-pump cardiac surgery between August 2012 and June 2018 at the Department of Cardiothoracic Surgery, Rigshospitalet, Copenhagen University Hospital, Denmark. Eligible patients were aged ≥18 years, with preoperative biobank samples available. Exclusion criteria included perioperative death (n = 4), preoperative dialysis dependency (n = 3), missing preoperative creatinine (n = 1), missing matching variables (n = 5), and suPAR values exceeding the assay's validated upper concentration of 22 ng/mL (n = 3). Blood samples were collected preoperatively and stored at −80 °C. Matrix tubes containing 150 μL of EDTA-plasma were retrieved and analysed at the Department of Clinical Biochemistry, Rigshospitalet, by experienced technicians blinded to clinical data. suPAR levels were measured using the suPARnostic® kit (ViroGates, Birkerod, Denmark), validated for concentrations between 0.6 and 22 ng/mL. We have previously performed internal validation of the assay and found an intra-assay coefficient of variation of 2.3%.^12^

We performed propensity score matching to balance baseline characteristics between groups and reduce selection bias. This statistical technique creates matched pairs that are similar in measured confounders, thereby mimicking some aspects of randomisation in observational data. We used a nearest-neighbour algorithm with a 2:1 ratio between controls (low suPAR, <4 ng/mL) and cases (high suPAR, ≥4 ng/mL) without calliper restrictions. Matching considered age, sex, diabetes, hypertension, and extracorporeal circulation duration, assessed by absolute standardised mean differences (ASMD).^13^ Preoperative patient characteristics and comorbidities, including recent myocardial infarction, were obtained from admission notes. Surgical procedure details were sourced from patient records, while extracorporeal circulation parameters were extracted from the local perfusionist database. Baseline kidney function was assessed using preoperative serum creatinine (collected within 30 days), with eGFR calculated using the Chronic Kidney Disease Epidemiology Collaboration (CKD-EPI 2021) equation.^14^ The primary analysis examined the relationship between suPAR levels and baseline eGFR in the matched cohort. Secondary analyses included AKI, defined by KDIGO (Kidney Disease: Improving Global Outcomes) criteria based on an absolute increase in serum creatinine within 48 h or a relative increase from baseline within 7 days, or initiation of kidney replacement therapy.^15^ This cohort was previously analysed for the association between suPAR and AKI using multivariable regression^11^; the present study instead focuses on the inverse association between preoperative suPAR and baseline eGFR.

### Porcine *ex vivo* model

To investigate suPAR's direct effects on renal vascular resistance under controlled conditions, eliminating systemic confounders while maintaining physiological perfusion parameters, we employed an *ex vivo* porcine kidney perfusion model. Fifteen female pigs (Danish Landrace x Yorkshire, ∼45 kg) were initially allocated to two groups: eight pigs to the control group and seven to the intervention group receiving recombinant human uPAR (800 μL, 10 μg/mL; 807-UK-100/CF, R&D Systems) after 30 min of *ex vivo* perfusion, aiming for a circuit concentration of 10 ng/mL. Two pigs from the intervention group were withdrawn due to malfunctioning vasodilating medication causing extremely low-flow conditions, and one pig from the control group was excluded due to sudden refractory haemodynamic instability during surgery. This resulted in final group sizes of 5 pigs (intervention) and 7 pigs (control) for analysis.

Pigs were anaesthetised using standard veterinary protocols with midazolam, medetomidine, ketamine, and butorphanol for premedication, followed by propofol induction and maintenance with propofol-fentanyl infusions. After midline laparotomy, the kidney with optimal vascular anatomy was identified. Following anticoagulation with 20,000 IU unfractionated heparin, 600 mL autologous whole blood was collected. The kidney was retrieved with vessels and ureter intact, cannulated using LifePort® straight cannulas, flushed with 200 mL room temperature 0.9% NaCl through the arterial line at a pressure of 100 cmH_2_O pressure, and immediately transported for *ex vivo* perfusion.

The perfusion circuit consisted of 1/4” and 3/8” PVC tubes, a hard-shell reservoir (VHK 11000; Maquet, Germany), a magnetic pump head (RF-32; Maquet, Germany) connected to a centrifugal pump unit (Rotaflow II; Maquet, Germany), and an oxygenator (Quadrox-I Neonatal; Maquet, Germany) (Supplementary Fig. S4, and Supplementary Movie S2). The kidney was placed in a customised polypropylene carrier, which minimised surface evaporation and heat loss while allowing prompt blood re-entry from outside the venous cannulation back into the circuit. The system was primed with 200 mL Ringer's acetate, 600 mL heparinised autologous whole blood, and 2 mL sodium bicarbonate 8.4%. Continuous infusions included verapamil (0.50 mg/h) for vasodilation and glucose-insulin solution (10 mL/h, 20 I.U. in 500 mL Glucose 10%) for glucose maintenance. The temperature within the oxygenator was kept constant at 37 °C using a water supply heater unit (HU 35; Maquet, Germany). A gas mixer (20090; Sechrist, USA) with inlets for air, oxygen, and carbon dioxide was used to maintain a partial pressure of arterial oxygen (PaO_2_) above 100 mmHg and a target partial pressure of arterial carbon dioxide (PaCO_2_) of approximately 40 mmHg. Perfusion pressure was measured as a side pressure on the LifePort® arterial cannula using an invasive pressure module (M1006B; Phillips, Holland). Blood flow was monitored using an ultrasonic flow probe (FBS 3/8″ x 3/32"; Maquet, Germany) connected to the centrifugal pump unit. Pump speed (rounds per minute) were gradually increased during the first 10 min of perfusion until reaching the target pressure of 80 mmHg at the arterial cannula entry. The experiment was terminated after 2.5 h of *ex vivo* perfusion.

Arterial and venous blood gases, plasma, urine, and kidney tissue biopsies were collected at predetermined intervals throughout the perfusion period (10 and 30 min, 1 h, and every 30 min thereafter). Plasma samples were analysed for creatinine, albumin, and neutrophil gelatinase-associated lipocalin (NGAL). Baseline suPAR levels were assessed using a porcine specific uPAR ELISA assays (MBS2501999; MyBioSource). Gene expression was analysed using qPCR with specific primers (Supplementary Table S6) for NGAL, endothelial nitric oxide synthase (eNOS), tumour necrosis factor-alpha (TNF-α), interleukin 1 beta (IL-1β), and interleukin 6 (IL-6). Kidney sections were evaluated by a blinded clinical pathologist using a semi-quantitative scoring system (Supplementary Material). Podocyte morphology was assessed using the podocyte exact morphology measurement procedure (PEMP) analysis, which quantifies filtration slit density (FSD) as a measure of podocyte health.^16^^,^^17^ Kidney sections were stained with antibodies targeting podocin and integrin α3 for filtration slit visualisation, and claudin 5 for detection of filtration slit reorganisation. Analysis was conducted by NIPOKA GmbH in a blinded manner, examining 20 randomly selected glomeruli from >200 glomeruli per section, with FSD calculated as slit membrane length per podocyte foot process area. For all immunofluorescence analyses, we used commercially available antibodies validated by manufacturers for the immunofluorescence applications in the respective species used.

### Mouse *in vivo* model

To visualise suPAR's cellular mechanisms and investigate tissue-specific effects, we used intravital multiphoton imaging in transgenic mice expressing genetically encoded calcium indicators. Male and female C57BL6/J mice (6–8 weeks old) were used in experiments. Sox2-Salsa6f mice were generated by crossing Sox2-Cre (RRID:IMSR_JAX:008454)^18^ and Salsa6f floxed (RRID:IMSR_JAX:031968)^19^ mice resulting in ubiquitous expression of the ratiometric fusion calcium indicator Salsa6f (tdTomato linked to GCaMP6f by a V5 epitope tag) in all renal cell types. Under continuous isoflurane anaesthesia (1–2%), mice were positioned on an inverted microscope stage for kidney or brain multiphoton microscopy imaging. For renal imaging, the exposed kidney was mounted in a coverslip-bottomed chamber bathed in normal saline as described previously.^20^^,^^21^ For brain imaging, a cranial window was created over the somatosensory cortex. Alexa Fluor 680-conjugated albumin was administered intravenously to label circulating plasma. Images were acquired using a Leica SP8 DIVE multiphoton system with 40 × water-immersion objective (numerical aperture (NA) 1.2) powered by a Chameleon Discovery laser at 960 nm (Coherent, Santa Clara, CA) and a DMI8 inverted microscope's external Leica 4 Tune spectral hybrid detectors (emission at 510–530 nm for GCaMP 6, at 580–640 nm for tdTomato, and at 680–740 for AF680) (Leica Microsystems, Heidelberg, Germany). Fluorescence images were collected in volume and time series (xyt, 526 ms per frame) for 3 min to measure intracellular calcium dynamics.

Animals received bolus injection of vehicle (50 μL 0.9% saline), suPAR (25, 50, 250, and 500 ng/animal; UPR-M52H3, ACROBiosystems, Newark, DE) in 50 μL of 0.9% saline, or angiotensin II (400 ng/kg in 50 μL 0.9% saline, EK-002-12CE, Phoenix Pharmaceuticals, Burlingame, CA) via the cannulated carotid artery alone or in combination with intraperitoneal injection of anti-uPAR antibody (500 μg/kg in 50 μL 0.9% saline). Five animals were allocated to each of the 7 groups (vehicle, suPAR 25, 50, 250, 500 ng, angiotensin II, suPAR (50 ng) + anti-uPAR antibody) via simple randomisation. The group allocations were available to all investigators.

Changes in calcium levels were quantified as normalised fluorescence intensity (F/F0) in regions of interest drawn over extraglomerular mesangium and vascular smooth muscle cells. Arteriole diameters were measured at baseline and maximum vasoconstriction.

Comprehensive details of all laboratory analyses and experimental procedures are available in the Supplementary Material.

### Sex, race and ethnicity as a biological variable

Sex was considered across all three sub-studies. In the clinical study, sex was defined by the binary biological sex recorded in the Danish Civil Registration System.^22^ The cohort included both male and female patients, and sex was incorporated into propensity score matching to ensure balanced representation. We note that the cohort was predominantly male, reflecting typical cardiac surgery demographics, which may limit generalisability to females. Race and ethnicity data were not collected in the clinical cohort. The study was conducted at a single centre in Denmark where the cardiac surgery population has limited racial and ethnic diversity. In the porcine *ex vivo* model, only females were used due to animal availability, while in the *in vivo* mouse model, both males and females were included with equal distribution across groups.

### Ethics

The clinical study was approved by the Research Ethics Committee for the Capital Region, committee B (H-18002379), with data storage approved by the Danish Data Protection Agency (VD-2018-15). The need for written informed consent for biobank analyses was waived by the Research Ethics Committee for the Capital Region. The *ex vivo* porcine protocol was approved by the Danish Animal Experimentation Inspectorate (2021-15-0201-01054), and the *in vivo* mouse studies were approved by the Institutional Animal Care and Use Committee at the University of Southern California (ACUC Protocols 10531 and 21492). Animal experiments are reported in compliance with ARRIVE guidelines.

### Statistics

Data normality was evaluated using Q–Q plots and the Shapiro–Wilk test. For the clinical study, between-group comparisons were conducted using chi-square tests for categorical outcomes and Wilcoxon rank-sum tests for continuous variables. The relationship between log2-transformed suPAR levels and baseline eGFR was analysed using Spearman's rank correlation coefficient.

For the porcine study, mixed-effects models evaluated time-dependent changes in continuous outcomes, incorporating fixed effects for time and group, and random effects for within-animal variability. Baseline comparisons and histopathology scores were assessed using Wilcoxon rank-sum tests. For the mouse study, between-group comparisons were conducted using the Kruskal–Wallis test with Dunn's post-hoc test or one-way ANOVA with Sidak's post-hoc test, depending on data distribution. *P* values from the clinical and porcine studies were adjusted for multiple testing using the false discovery rate method (Benjamini-Hochberg).^23^ A two-sided adjusted *P* value < 0.05 was considered statistically significant. Analyses were performed using R version 4.4.2 (R Core Team 2024) and GraphPad Prism version 10.4.1 (GraphPad Software, Boston, USA). Propensity score matching was implemented using the MatchIt package^24^ in R.

For both animal studies, formal sample size calculations were not performed due to the novel application of these models to assess suPAR's vascular effects and absence of prior effect size data for this specific intervention. Sample sizes were based on previous similar *ex vivo* studies with comparable group sizes^25^^,^^26^ or followed institutional practice.

### Role of funders

The funders had no role in study design, data collection, data analyses, interpretation, or writing of report.

## Results

### Baseline kidney function and AKI risk in cardiac surgery patients

**Fig. 1:**
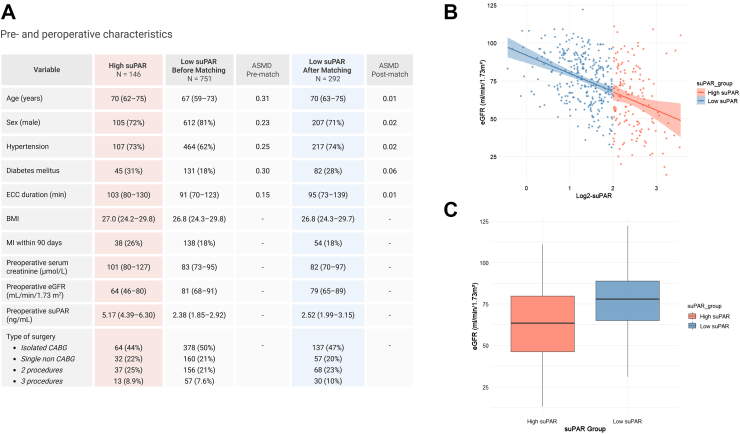
Preoperative suPAR levels and kidney function in cardiac surgery patients. (*A*) Baseline and perioperative characteristics comparing patients with high suPAR (≥4 ng/mL, n = 146) vs matched (n = 292) and unmatched (n = 751) controls with low suPAR (<4 ng/mL). Values shown as median (25th–75th percentiles) or n (%). ASMD values are only shown for variables included in the propensity score matching model. (*B*) Correlation between log2-transformed pre-operative suPAR and eGFR in high suPAR (red) and propensity-matched low suPAR (blue) groups, with 95% confidence intervals. (*C*) Box plots of pre-operative eGFR distribution in both groups, displaying median, 25th–75th percentiles, and range. Abbreviations: ASMD, absolute standardised mean difference; BMI, body mass index; CABG, coronary artery bypass grafting; ECC, extracorporeal circulation; eGFR, estimated glomerular filtration rate (CKD-EPI 2021); MI, myocardial infarction; suPAR, soluble urokinase plasminogen activator receptor.

The clinical population consisted of 897 cardiac surgery patients with a median age of 67 years (59–73), of whom 80% (717/897) were male. Hypertension was present in 64% (571/897) and diabetes in 20% (180/897) of patients. Propensity score matching paired 146 patients with elevated suPAR levels (≥4 ng/mL) with 292 controls in a 1:2 ratio. The matching process substantially improved covariate balance, as evidenced by the reduction in ASMD for all selected baseline characteristics to values well below the recommended threshold of 0.10 (Fig. 1). The median value of suPAR was 5.17 (4.39–6.30) ng/mL and 2.52 (1.99–3.15) ng/mL for the matched high and low suPAR group, respectively (Fig. 1). Preoperative eGFR was lower in the high suPAR group, with a mean difference of 14.5 mL/min/1.73 m^2^ (95% CI: 18.5 to 10.4) compared to the low suPAR group. Within each group, there was a significant inverse correlation between suPAR levels and baseline kidney function (low suPAR: ρ = −0.360, *P* < 0.001; high suPAR: ρ = −0.202, *P* = 0.015).

In the matched cohort analysis, patients with high suPAR had a significantly higher rate of AKI compared to those with low suPAR (56% [81/146] vs 33% [95/292]; relative risk 1.71, 95% CI 1.37–2.12, *P* < 0.001). This was reflected in significantly higher peak postoperative creatinine levels and greater absolute increase in creatinine in the high suPAR group (Supplementary Table S1). Furthermore, patients with high suPAR had a significantly higher need for kidney replacement therapy (8.2% [12/146] vs 2.4% [7/292]; relative risk 3.43, 95% CI 1.38–8.52, *P* = 0.012).

### suPAR-induced increase in renal vascular resistance

A total of 12 pigs were included for analysis with comparable pre-surgery weights (45.7 (44.3–46.3) kg vs 45.1 (43.3–45.1) kg) and perioperative laboratory parameters (Supplementary Table S2). The median time of kidney ischaemia until reperfusion was similar between groups (51 min), and baseline suPAR concentrations were low in both the control and suPAR groups (0.30 vs 0.19 ng/mL, *P* = 0.21) (Supplementary Fig. S1). Physiological parameters remained within prespecified targets throughout perfusion (Supplementary Fig. S2 and Table S3).

**Fig. 2:**
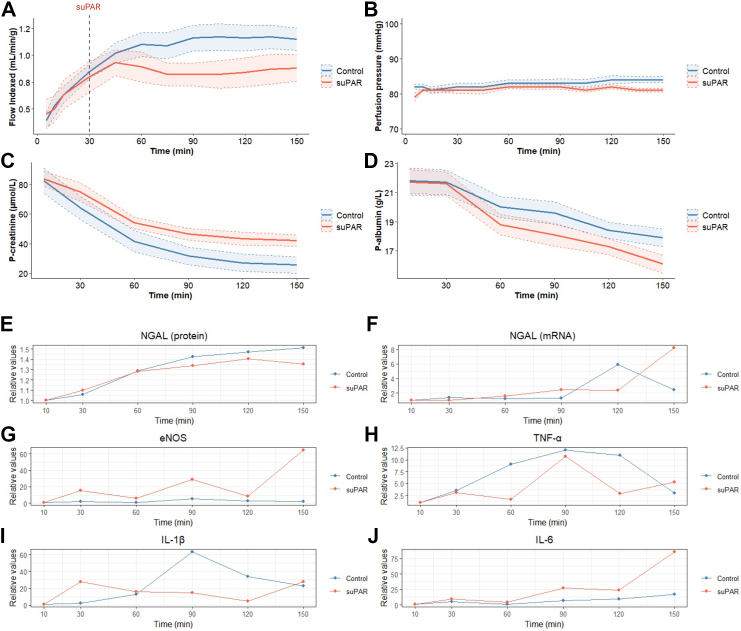
Physiological parameters and molecular markers during *ex vivo* perfusion. (*A*) Weight-indexed blood flow, (*B*) perfusion pressure, (*C*) plasma creatinine (P-creatinine), and (*D*) plasma albumin (P-albumin) comparing control and suPAR groups. Note the immediate drop in kidney blood flow after suPAR infusion (dashed line), followed by sustained reduction despite unchanged perfusion pressures between groups. Data are expressed as mean ± SEM (n = 5 suPAR group, n = 7 control group). Inflammatory and kidney injury biomarkers in control vs suPAR groups: (*E*) Neutrophil gelatinase-associated lipocalin (NGAL) protein measured by ELISA in the perfusion circuit, (*F*) gene expression of NGAL, (*G*) endothelial nitric oxide synthase (eNOS), (*H*) tumour necrosis factor-alpha (TNF-α), (*I*) interleukin 1 beta (IL-1β), and (*J*) interleukin 6 (IL-6). Gene expression was quantified by qPCR, normalised to ribosomal protein L4 (RPL4), and expressed relative to 10-min baseline values.

Blood flow gradually increased within the first 45 min of *ex vivo* perfusion until it reached a plateau of around 80 mL/min for the suPAR group (targeting 10 ng/mL circulating concentration) and 120 mL/min for the control group (*P* = 0.003) (Fig. 2A and Supplementary Fig. S2A). This disparity was primarily driven by reduced flow in the suPAR group shortly after administration, despite comparable perfusion pressures between groups (*P* = 0.13) (Fig. 2B).

We observed a gradual decrease in plasma creatinine and albumin throughout the perfusion period (Fig. 2C and D). The suPAR group showed higher urinary albumin and lower creatinine excretion, though these differences were not statistically significant after correction for multiple testing (Supplementary Table S3). Urinary output showed marked variability, with median values of 2.3 (1.3–3.1) mL/h in the suPAR group vs 13.9 (8.1–18.4) mL/h in controls (Supplementary Fig. S3A). Due to substantial variations both between and within animals, urine parameters were not used for further group comparisons. However, analysis of available samples confirmed that the declining plasma creatinine and albumin levels were due to continuous urinary excretion rather than haemodilution (Supplementary Fig. S3B and C).

**Fig. 3:**
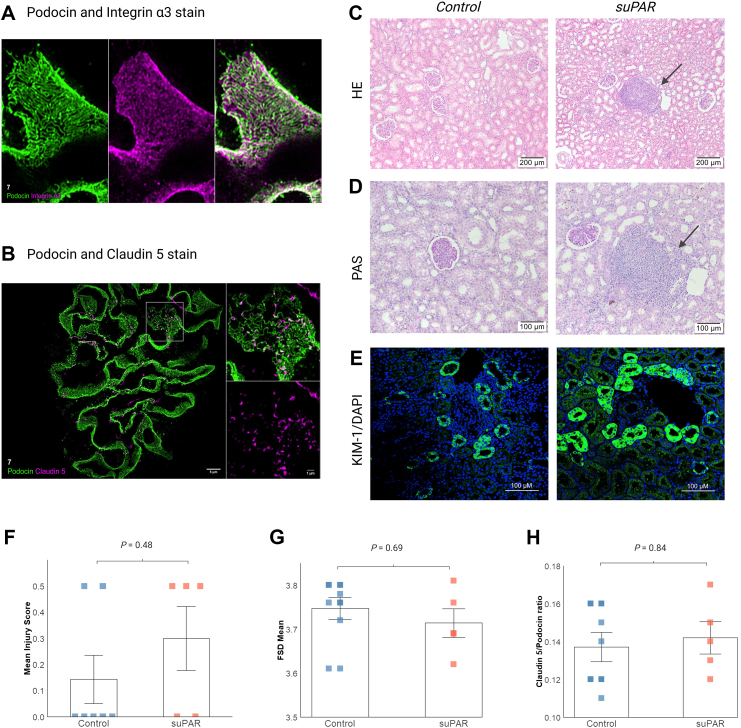
Structural analysis of kidney tissue after *ex vivo* perfusion. (*A*) Immunofluorescence microscopy of podocin (green) and integrin α3 (magenta) with merged image. (*B*) Immunofluorescence microscopy of podocin (green) and claudin 5 (magenta) with high-magnification inset of a single glomerulus. (*C*) Representative haematoxylin-eosin (HE) and (*D*) Periodic acid-Schiff (PAS) stained sections from control and suPAR-treated kidneys. Arrows indicate focal interstitial inflammatory infiltrates. (*E*) Representative immunofluorescence images showing Kidney Injury Molecule-1 (KIM-1, green) expression and DAPI nuclear staining (blue) in control and suPAR-treated kidneys. KIM-1 expression is localised to proximal tubules, with more intense and widespread staining apparent in suPAR-treated tissue. (*F*) Histopathological injury scores based on blinded evaluation of PAS and HE stained sections using a standardised scoring system. Quantitative analyses comparing control and suPAR groups in podocyte exact morphology measurement procedure (PEMP) analysis: (*G*) Filtration slit density (FSD) measuring podocyte foot process effacement and (*H*) claudin 5/podocin ratio assessing filtration barrier integrity, both analysed from 20 randomly selected glomeruli per kidney. Individual data points and mean ± SEM are presented (n = 5 suPAR group, n = 7 control group). Statistical analysis by Wilcoxon rank-sum test with Benjamini-Hochberg correction^23^ for multiple testing.

Plasma NGAL levels exhibited a significant temporal increase throughout the *ex vivo* perfusion but without any significant temporal difference between groups (*P* = 0.55) (Fig. 2E). Furthermore, no temporal difference between groups was observed regarding gene expression of NGAL (*P* = 0.21), eNOS (*P* = 0.12), TNF-α (*P* = 0.45), IL-1β (*P* = 0.29) and IL-6 (*P* = 0.90) in kidney tissues (Fig. 2 F–J). Histopathological evaluation revealed a tendency towards higher interstitial inflammation in suPAR-treated pigs. However, pathological characteristics were generally discrete without significant differences in injury scores (*P* = 0.48) (Fig. 3 C, D and F). Immunofluorescence analysis revealed increased Kidney Injury Molecule-1 (KIM-1) expression in suPAR-treated kidneys compared to controls, with more intense staining and broader distribution pattern in proximal tubules, suggesting early tubular stress responses (Fig. 3E). PEMP analysis revealed no significant difference in FSD values between the two groups (*P* = 0.69). Mean FSD values were high and similar in both suPAR-treated (3.75 μm) and control pigs (3.71 μm), indicating a lack of significant podocyte effacement (Fig. 3G). Similarly, the Claudin 5/Podocin ratio was similar between groups (*P* = 0.84), suggesting no difference in the ongoing reorganisation of the filtration slit (Fig. 3H).

### Effect of suPAR on renal vasculature and extra-glomerular mesangial cells

**Fig. 4:**
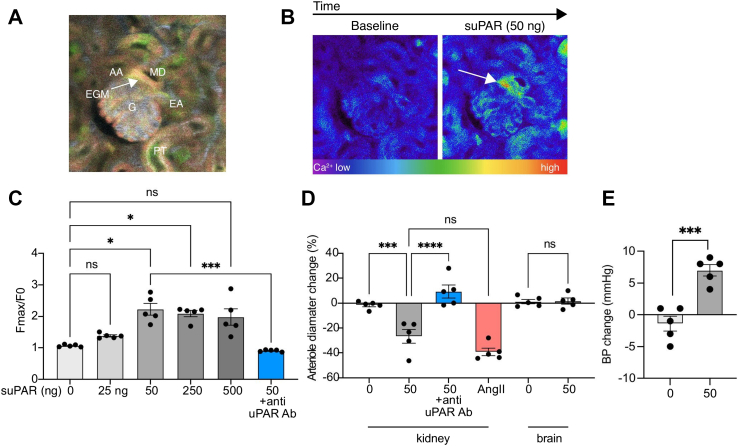
Acute effects of suPAR on renal haemodynamics, calcium signalling, and blood pressure in Sox2-Salsa6f mice. (*A*) Intravital multiphoton microscopy image of a control healthy Sox2-Salsa6f mouse kidney glomerulus (G) with the afferent (AA) and efferent (EA) arterioles, extraglomerular mesangium (EGM, arrow), and macula densa (MD). Note the highest intensity of the genetically encoded calcium reporter GCaMP6f (green) and the calcium-insensitive tdTomato (red) in the MD compared to all kidney cell types. The circulating plasma was labelled by intravenous injected albumin–Alexa Fluor 680 (grey). (*B*) Representative pseudocolour GCaMP6f fluorescence intensity images of the same tissue segment at baseline (left) and 5 s after suPAR injection (right, peak effect). Note the most robust calcium increase in cells of the EGM at the base of the macula densa in response to acute suPAR injection (ic. 50 ng in 50 μL 0.9% saline) indicated by the appearance of yellow-red pseudocolour labelling (arrow). (*C*) Statistical summary of the dose-dependent effects of acute suPAR on EGM calcium (0, 25, 50, 250, 500 ng in 50 μL 0.9% saline) with or without pretreatment with anti-uPAR antibody (ip. 500 μg/kg in 50 μL 0.9% saline). (*D*) Statistical summary of the acute vasoconstrictor effects of suPAR (0 or 50 ng in 50 μL 0.9% saline) in the mouse kidney and brain, and of angiotensin II (400 ng/kg, ic., AngII) and anti-uPAR pretreatment (ip. 500 μg/kg in 50 μL 0.9% saline) in the kidney. (*E*) Statistical summary of the acute effects of suPAR (ic. 50 ng in 50 μL 0.9% saline) on systolic blood pressure. Data are expressed as mean ± SEM (n = 5 mice per group), ns: not significant, ∗ –∗∗∗∗: *P* < 0.05–0.0001 using Kruskal–Wallis test with Dunn's multiple comparison test (C), or separate one-way ANOVA with Sidak's multiple comparison test (D), or unpaired t test (E). Shapiro–Wilk test was used to test for normality.

To study the effects of acute suPAR administration on cell physiology in 4 dimensions in comparative cell mode (cc4DP), Sox2-Salsa6f mice and *in vivo* multiphoton microscopy imaging was used. The ubiquitous expression of the genetically encoded calcium reporter GCaMP6f/tdTomato in Sox2-Salsa6f mice allowed quantitative visualisation of the changes in intracellular Ca^2+^ ([Ca^2+^]_i_) with high spatial and temporal resolution in all kidney cell types. The highest tdTomato expression in macula densa cells, combined with the clear visualisation of the glomerulus, afferent and efferent arterioles, enabled precise identification of microanatomical regions, including the vascular pole and the extraglomerular mesangium (EGM) (Fig. 4A).

While the injection of vehicle control had no effect, acute systemic injections of suPAR into the cannulated carotid artery (ic.) in bolus caused instantaneous and robust elevations in intracellular calcium levels in contractile cells, including the EGM, afferent and efferent arteriole vascular smooth muscle cells, and the intraglomerular mesangium (Fig. 4B and Supplementary Movie S1). In addition, cells of the proximal tubule and the macula densa were also responsive. The most robust effect was observed in the EGM and was dose-dependent (Fig. 4 B and C).

Furthermore, acute ic. administration of suPAR resulted in immediate glomerular haemodynamic responses, including significant afferent arteriole vasoconstriction (Fig. 4D). Notably, the magnitude of suPAR-induced afferent arteriole vasoconstriction was comparable to the effect of moderate doses of the classic vasoconstrictor angiotensin II (ic. 400 ng/kg) (Fig. 4D).

The suPAR-induced elevations in [Ca^2+^]_i_ and haemodynamic responses were mainly expressed in the renal vasculature and were completely prevented by pretreatment with anti-uPAR antibody (500 μg/kg in 50 μL 0.9% saline) (Fig. 4 C–D). Similar resistance arterioles in the brain did not produce calcium elevations or vasoconstriction in response to suPAR (Fig. 4D). On the systemic level, suPAR caused small but significant blood pressure elevations (Fig. 4E).

To assess the corresponding systemic suPAR distribution, we measured serum and urine levels in C57BL/6j (RRID:IMSR_JAX:000664) mice (n = 9) after injection of 50 ng recombinant suPAR (UPR-M52H3, ACROBiosystems, Newark, DE). Assay validation showed that the bioactive concentration of the recombinant suPAR was approximately one-fifth of the labelled dose, likely due to reduced immunoreactivity relative to the uPAR ELISA kit (R&D Systems) standard, or partial protein degradation. Accordingly, the 50 ng injection corresponded to an effective dose of ∼10 ng, achieving serum concentrations that rose from ∼1 ng/mL to ∼3 ng/mL at 24 h (Supplementary Fig. S5). Urine suPAR concentrations showed a similar progressive increase over 24 h, confirming both systemic circulation and renal clearance of the administered suPAR. These circulating levels, along with the suPAR concentrations used in our *ex vivo* porcine model, correspond to elevated physiological ranges observed in high-risk clinical populations.

## Discussion

This study identified a mechanism by which suPAR directly modulates renal vascular tone. In a cohort of cardiac surgery patients, elevated preoperative suPAR levels were associated with lower preoperative eGFR and increased occurrence and severity of postoperative AKI, even after adjustment for key confounders through propensity score matching. Mechanistic insights supporting these findings were derived from *ex vivo* porcine and *in vivo* mouse models, demonstrating that suPAR induces calcium-dependent vasoconstriction in renal contractile cells, thereby impairing renal blood flow.

Reduced blood flow in the kidney is a well-established precursor to AKI.^27^ While systemic haemodynamics often contribute to compromised renal perfusion, alterations in intrarenal vascular tone play a key role in regional disturbances. Using intravital multiphoton imaging, we observed that suPAR triggers robust calcium responses in renal contractile cells, particularly in EGM and vascular smooth muscle cells. This cellular calcium increase led to significant vasoconstriction comparable to moderate doses of angiotensin II.

Our *ex vivo* porcine model provided further mechanistic support, as suPAR administration immediately reduced renal blood flow, despite stable perfusion pressures. This underscores the direct effects of suPAR on increased vascular resistance. Notably, the calcium-dependent vasoconstriction observed in EGM cells strategically located at the vascular pole points to a distinct mechanism by which suPAR affects glomerular perfusion. EGM cells enable precise regulation of glomerular perfusion via contractile properties and interactions with afferent and efferent arterioles. This strategic positioning enables precise local haemodynamic control.

The kidney specificity of suPAR's vasoconstrictor effects is demonstrated by the absence of calcium responses or vasoconstriction in comparable brain resistance arterioles (Fig. 4D). While modest systemic blood pressure elevation was observed, this was minimal compared to the pronounced renal effects, supporting predominantly kidney-specific vasoconstrictor mechanisms.

Notably, the reduction in renal blood flow in our porcine model was observed despite continuous infusion of high-dose Verapamil, a potent voltage-gated L-type calcium channel blocker. This suggests that suPAR's effects on calcium signalling may involve alternative pathways, such as release from intracellular stores or localised calcium signalling domains, rather than purely through voltage-gated L-type calcium channels. Although this study did not directly investigate integrins, previous research has demonstrated that suPAR can form high-affinity interactions with αvβ3 integrins.^28^ Specifically, αvβ3 integrins are known to regulate rapid calcium mobilisation through well-characterised pathways involving focal adhesion kinase activation and tyrosine kinase signalling.^29^ This localised mechanism may explain the tissue-specific effects observed in our studies and offer an alternative perspective on how suPAR contributes to kidney dysfunction in acute settings beyond its well-documented effects in CKD.^28^^,^^30^^,^^31^

Interestingly, we observed an apparent discrepancy between the magnitude of afferent arteriole diameter reduction (∼30% in mice) and the severity of functional impairment in our *ex vivo* model. This disparity also reflects the difference between our clinical findings, where suPAR was strongly associated with AKI development, and our experimental models, where the vascular effects could not be translated to measurable functional decline over the 2.5-h observation period. Our experimental models were designed to detect short-term vascular changes, whilst clinical AKI develops over hours to days with multiple contributing systemic factors absent from our controlled *ex vivo* system. This pattern suggests that suPAR's vascular effects may be particularly detrimental when combined with other AKI risk factors. In cardiac surgery, for example, cardiopulmonary bypass alone increases renal vascular resistance by approximately 20%.^32^ The additive effect of elevated suPAR levels in such clinical contexts may explain why preoperative suPAR strongly predicts postoperative AKI severity in our clinical study. While the precise molecular mechanisms underlying suPAR-induced vasoconstriction require further investigation, our findings suggest that suPAR may exert a pre-sensitising effect on renal vasculature.

These findings raise a fundamental question: does suPAR merely reflect reduced kidney function or actively drive kidney injury? Population-based studies suggest the latter, with suPAR identified as a predictor of future eGFR decline independent of baseline kidney function.^33^^,^^34^ This is further supported by a recent large-scale proteomic study demonstrating robust associations between elevated suPAR levels and CKD development.^35^ Our findings provide mechanistic insight into how suPAR might influence kidney function differently in acute vs chronic settings. While previous studies have shown suPAR-induced podocyte structural changes in CKD models,^28^^,^^30^ our PEMP analysis revealed no significant differences in podocyte morphology following acute suPAR exposure, with filtration slit density and Claudin 5/Podocin ratios remaining unchanged. The immediate vascular response to suPAR and preserved podocyte structure suggest vasoconstriction represents a crucial early mechanism of suPAR's effects on kidney function. In contrast, podocyte-mediated effects may require more prolonged exposure or different pathological conditions.

This study has several limitations. The retrospective, single-centre design of the patient cohort restricts both causal inference and generalisability. Although propensity score matching helped balance measured confounders between groups, it cannot replace randomised controlled trials, as unmeasured variables may still have influenced the observed inverse relationship between suPAR and eGFR. Our experimental animal models do not accurately reflect clinical scenarios, as suPAR does not typically undergo sudden increases in patients.^36^^,^^37^ The *ex vivo* porcine perfusion model, while providing controlled conditions to isolate direct renal effects of suPAR, lacks systemic physiological responses and neurohumoral regulation, and therefore does not encompass the full complexity of renal physiology. Furthermore, we could not confirm whether we achieved target concentrations of suPAR in this model, as the available porcine suPAR ELISA assay did not cross-react with recombinant human suPAR. While no differences in NGAL levels were detected, we observed increased KIM-1 expression in proximal tubules of suPAR-treated kidneys. However, this finding should be interpreted with caution as it is based on qualitative assessment of representative images without quantitative analysis. This pattern of early KIM-1 upregulation without concurrent NGAL elevation likely reflects the temporal evolution of injury, where vascular changes and initial tubular stress responses precede more extensive cellular damage. Previous studies have shown that alterations in ischaemia-reperfusion injury evolve over hours before manifesting as measurable injury markers.^38^ Finally, while our stepwise translational design provided evidence that suPAR increases afferent arteriole vasoconstriction, the net impact on overall renal blood flow and AKI occurrence cannot be definitively determined from these studies alone. Although afferent arteriolar vasoconstriction might theoretically redirect blood flow toward peritubular capillaries if accompanied by relative efferent arteriolar dilation or less severe efferent constriction, our clinical data—showing increased AKI rates in patients with elevated suPAR—suggest that any such compensatory redistribution is insufficient to offset the overall reduction in renal perfusion. Future studies should examine whether sustained exposure to suPAR leads to longer-term effects, such as structural alterations in podocytes, progression to tubular injury, or activation of inflammatory pathways that may exacerbate kidney dysfunction over time. In addition, further research should explore the molecular basis of suPAR's tissue-specific vascular effects, including comparative analyses of receptor expression patterns, signalling pathway differences, and local microenvironmental factors that may determine vascular responsiveness across different organ systems.

In conclusion, this study identifies calcium signalling in renal contractile cells as a critical mechanism by which suPAR impairs kidney function. These findings provide insights into the pathophysiology of AKI and suggest potential therapeutic targets for mitigating suPAR's harmful effects on renal vasculature.

## Contributors

Conceptualisation, SBR, CW, JPP, PS, JR, and HBR; Methodology, SBR, RBL, GG, KM, CB, LL, SRO, CW, JPP, PS, JR, and HBR; Investigation, SBR, RBL, GG, KM, CW, JPP, PS, and HBR; Visualisation, SBR, GG, KM, CW, JPP, and PS; Funding acquisition, SBR, JPP, PS, JR and HBR; Project administration, SBR, JPP, PS, JR, and HBR; Writing—original draft, SBR, GG, and CW; Writing—review & editing, SBR, RBL, GG, KM, CB, LL, SRO, CW, JPP, PS, JR, and HBR. All authors read and approved the final version of the manuscript. SBR and HBR accessed and verified all clinical data, SBR and PS accessed and verified all *ex vivo* perfusion data, and GG and JPP accessed and verified all *in vivo* mouse data; each author takes responsibility for the integrity and accuracy of their respective data.

## Data sharing statement

All data supporting the findings of this study are included in the article, Supplementary Material, and Data S1 file. Further data sharing requests should be directed to sebastian.buhl.rasmussen@rsyd.dk.

## Declaration of interests

JR is a cofounder and shareholder of Walden Biosciences, a biotechnology company that develops kidney-protective therapies. JPP has received research support from Travere Therapeutics Inc. and honoraria for lectures from Eli Lilly and Pfizer. JPP and GG are co-founders of Macula Densa Cell LLC, a biotechnology company that develops therapeutics to target macula densa cells for a regenerative treatment for chronic kidney disease. Macula Densa Cell LLC has a patent entitled “Targeting macula densa cells as a new therapeutic approach for kidney disease” (US patents 10828374 and 11318209). SBR has received a postdoctoral grant from the Lundbeck Foundation. GG has received the American Society of Nephrology Carl W. Gottschalk Transition to Independence grant. The other authors declare no competing interests.

## Appendix A Supplementary data

Supplementary Figures and Tables

Data set
