# Supplementary material for: Soluble urokinase receptor is a kidney-specific vasoconstrictor
Source: eBioMedicine. 2025 Nov 3;121:106012. doi: 10.1016/j.ebiom.2025.106012 (PMC12629921; doi:10.1016/j.ebiom.2025.106012)
Supplement: Supplementary Figures and Tables [file mmc1.pdf]

Supplementary Materials

Soluble urokinase receptor is a kidney-specific vasoconstrictor

Sebastian Buhl Rasmussen*, Rasmus Bo Lindhardt, Georgina Gyarmati, Kirsten Madsen, Claus Bistrup, Lars Lund, Sisse Rye Ostrowski, Changli Wei, Janos Peti-Peterdi, Per Svenningsen, Jochen Reiser*, Hanne Berg Ravn

*Corresponding authors: Sebastian Buhl Rasmussen and Jochen Reiser
Email: sebastian.buhl.rasmussen@rsyd.dk, jreiser@utmb.edu

This PDF file includes:

Supporting information
Figures S1 to S5
Tables S1 to S6
Legends for Movies S1 to S2
Legends for Datasets S1
Supplementary Materials references

Other supporting materials for this manuscript include the following:

Movies S1 to S2
Datasets S1

Supporting information

Histopathological scoring system for porcine kidney injury assessment. Paraffin-fixed tissue sections from the renal cortex and medulla were stained with Periodic acid–Schiff (PAS) and haematoxylin and eosin (HE). The kidney sections were then assessed by a clinical pathologist (KM) unaware of the study allocation. For this purpose, we utilised a semi-quantitative scoring system consisting of the following histopathological criteria:

1. Tubular vacuolisation
2. Tubular dilatation and loss of brush border
3. Cortical tubular necrosis
4. Medullary tubular necrosis
5. Interstitial inflammation

Each of these categories received a score based on the degree kidney cortical involvement:

0 = No injury
0.5 = 1-5% involvement
1 = 5-10% involvement
2 = 10-25% involvement
3 = 25-50% involvement
4 = >50% involvement

Supplementary materials and methods

Anaesthesia and surgical procedure for *ex vivo* model. The pigs used for the *ex vivo* experimental model were transferred from a local breeding herd with highest health status according to the SPF-Denmark programme (Kokkenborg ApS, DK-5771 Stenstrup, Red SPF) to the research facility on the day of the experiment. The animals were co-transported in pairs and concurrently anaesthetised in the morning to minimise environmental stress. All pigs were premedicated with an intramuscular injection containing midazolam 0.25 mg/kg (Midazolam Hameln; Hameln Pharma gmbh, Germany), medetomidine 0.03 mg/kg (Cepetor Vet; ScanVet Animal Health A/S, Denmark), ketamine 5 mg/kg (Ketaminol Vet; MSD Animal Health, Denmark), and butorphanol 0.2 mg/kg (Butomidor Vet; Salfarm Danmark A/S, Denmark). After achieving full muscle relaxation, the animals were transferred to the operating bed, where intravenous access was established while oxygen saturation was monitored. General anaesthesia was induced using a 1.5-2 mg/kg propofol bolus (Proposure; ScanVet Animal Health A/S, Denmark), followed by orotracheal intubation and mechanical ventilation. Anaesthesia was maintained with continuous infusions of propofol 10 mg/kg/h and fentanyl 0.2-0.6 mg/kg/min (Fentanyl Hameln; Hameln Pharma gmbh, Germany). Continuous perioperative monitoring included non-invasive blood pressure, heart rate, 3-point electrocardiogram, oxygen saturation, and capnography. Pigs were supplemented with continuous intravenous Ringer's acetate (Fresenius Kabi, Sweden) and received a Foley bladder catheter.

After established anaesthesia, we performed a midline laparotomy for localisation of the aorta, vena cava, and both kidneys. The kidney with the most suitable vessel anatomy was identified. A 6 Fr sheath (Terumo Europe N.V., Belgium) was inserted in the abdominal aorta using an 18 G introducer needle and guidewire. Next, the pig was anticoagulated by administration of 20,000 I.U. unfractionated heparin (Ambros I/S, Denmark). A sterile blood bag (1000 mL, Maquet) with 10,000 I.U. unfractionated heparin was then attached to the sheath to collect 600 mL of whole blood. After blood collection, a ligature was placed on the renal artery at the aortic branching, and the vena cava was ligated cranially and caudally from where the renal vein(s) enter. Then, the kidney including vessels and ureter was retrieved, and the pig was euthanised with i.v. pentobarbital 180 mg/kg (Exagon Vet; Salfarm Danmark A/S, Denmark).
Next, the renal artery and part of the vena cava or renal vein (depending on vessel anatomy) were cannulated using a LifePort® straight cannula (Organ Recovery Systems, Belgium), and the ureter was cannulated using a CH 5 nasogastric tube. The kidney was then flushed with room temperature 200 mL NaCl 0.9% (Fresenius Kabi, Sweden) through the arterial line at a pressure of 100 cmH_2O. Finally, the

kidney was promptly transported directly to the neighbouring research facility, where the kidney was weighed, and the ischaemia time was recorded before startup of *ex vivo* kidney perfusion.

***Ex vivo* kidney perfusion setup.** The perfusion circuit consisted of 1/4” and 3/8” PVC tubes, a hard-shell reservoir (VHK 11000; Maquet, Germany), a magnetic pump head (RF-32; Maquet, Germany) connected to a centrifugal pump unit (Rotaflow II; Maquet, Germany), and an oxygenator (Quadrox-I Neonatal; Maquet, Germany) (Fig. S4, and Movie S2). The kidney was placed in a customised polypropylene carrier, which minimised surface evaporation and heat loss while allowing prompt blood re-entry from outside the venous cannulation back into the circuit. The system was primed with 200 mL of Ringer's acetate (Fresenius Kabi, Sweden), 600 mL heparinised autologous whole blood, and 2 mL Sodium Bicarbonate 8.4% (Monico spa, Italy). The system was continuously infused with verapamil 0.50 mg/h (Isoptin; Mylan, USA) and glucose-insulin 10 mL/h (20 I.U. (Novo Nordisk, Denmark) in 500 mL Glucose 10% (Fresenius Kabi, Sweden)) to maintain sufficient vasodilation and glucose level in the circuit. The temperature within the oxygenator was kept constant at 37˚C using a water supply heater unit (HU 35; Maquet, Germany). A gas mixer (20090; Sechrist, USA) with inlets for air, oxygen, and carbon dioxide was used to maintain a partial pressure of arterial oxygen (PaO_2) above 100 mmHg and a target partial pressure of arterial carbon dioxide ($PaCO_2$) of approximately 40 mmHg. Perfusion pressure was measured as a side pressure on the LifePort® arterial cannula using an invasive pressure module (M1006B; Phillips, Holland). Blood flow was monitored using an ultrasonic flow probe (FBS 3/8" x 3/32"; Maquet, Germany) connected to the centrifugal pump unit. Rounds per minute were gradually increased during the first 10 minutes of perfusion until reaching the target pressure of 80 mmHg at the arterial cannula entry. The experiment was terminated after 2.5 hours of *ex vivo* perfusion.

***Ex vivo* model sampling and laboratory analysis details.** Punch biopsies (3 mm) were immediately frozen at -80˚C after collection. Plasma was obtained by centrifuging whole blood at 3000 rpm (4˚C, 10 minutes) and stored at -80˚C. Post-experiment kidney tissues were formalin-fixed for up to 24 hours before transfer to phosphate-buffered saline with 0.05% sodium azide. Plasma creatinine and albumin were analysed using absorption photometry (Cobas 8000, Roche Diagnostics; validated ranges: 5-2700 µmol/L and 2-60 g/L). Blood gases were analysed on-site (ABL90 FLEX, Radiometer) for pH, base excess, PaO2, PaCO2, saturations, haemoglobin, electrolytes, glucose, and lactate.
ELISA assays included: uPAR kit (MBS2501999; MyBioSource, detection range: 62.5-4000 pg/mL) and NGAL in pig plasma (detection range: 4-400 pg/mL). Gene expression was analysed using qPCR with specific primers for Neutrophil gelatinase-associated lipocalin (NGAL), endothelial nitric oxide synthase (eNOS), Tumour necrosis factor-alpha (TNF-α), Interleukin 1 beta (IL-1β), and Interleukin 6 (IL-6), normalised to Ribosomal Protein L4 (RPL4), with all measurements performed in duplicate.
For the podocyte exact morphology measurement procedure (PEMP), kidney sections were stained with antibodies targeting Podocin and Integrin α3 for filtration slit and foot process visualisation. Claudin 5 staining enabled detection of filtration slit reorganisation. Analysis involved 20 randomly selected glomeruli from >200 glomeruli per section, conducted blind by NIPOKA GmbH, with filtration slit density (FSD) calculated as slit membrane length per podocyte foot process area.

***In vivo* multiphoton microscopy.** Under continuous anaesthesia (Isoflurane 1–2% inhalant via nosecone), mice were placed on the stage of the inverted microscope for kidney or brain MPM imaging. For imaging renal microvessels, the exposed kidney was mounted in a coverslip-bottomed chamber bathed in normal saline as described previously[1,2]. For MPM imaging of brain arterioles, the forelimb/hindlimb region of the somatosensory cortex was made accessible via a cranial window[3]. Briefly, animals were continuously anaesthetised with isoflurane and fixed in a stereotaxic frame. After surgical scalp removal, a circular cranial window was drilled and filled with 2% low melt agarose (Sigma) in artificial cerebrospinal fluid and covered with a round glass coverslip. Body temperature was maintained with a homeothermic blanket system (Harvard Apparatus). Alexa Fluor 680-conjugated albumin (Thermo Fisher, Waltham, MA) was administered iv. by retro-orbital injections to label the circulating plasma (30 µL iv. bolus from 10 µg/mL stock solution). The images were acquired using a Leica SP8 DIVE multiphoton confocal fluorescence imaging system with a 40× Leica water-immersion objective (numerical aperture (NA) 1.2) powered by a Chameleon Discovery laser at 960 nm (Coherent, Santa Clara, CA) and a DMI8 inverted microscope’s external Leica 4Tune spectral hybrid detectors (emission at 510–530 nm for GCaMP6, at 580–640 nm for tdTomato, and at 680–740 for AF680) (Leica Microsystems, Heidelberg, Germany). The potential toxicity

of laser excitation and fluorescence to the cells was minimised by using a low laser power and high scan speeds to keep total laser exposure as minimal as possible. Fluorescence images were collected in volume and time series (xyt, 526 ms per frame) with the Leica LAS X imaging software and using the same instrument settings (laser power, offset, gain of both detector channels). The strong, positive, cell-specific Salsa6f signal (GCaMP6f - tdTomato fluorescence) and high-resolution MPM imaging allowed for easy identification of single cell bodies and anatomical structures.

Quantification of Salsa6f fluorescence intensity and vascular diameter. Optical sections were selected in which a glomerulus with its vascular pole, macula densa and extraglomerular mesangium, and the terminal segments of the afferent and efferent arteriole in the kidney, and long segments of cerebral microvessels in the brain were clearly visible. Time (xyt) series with 1 frame per 526 milliseconds were recorded for 3 minutes to measure $[Ca^{2+}]_i$ dynamics. The highest positive signal of tdTomato in Sox2-Salsa6f mice and high-resolution MPM imaging allowed for easy identification of the macula densa as described before[4]. EGM was defined between the base of the macula densa and the terminal segments of the afferent and efferent arterioles. For the quantification of changes in mean Salsa6f ratio, ROIs were drawn closely over the EGM and the changes in normalised F/F0 (GCaMP6f/tdTomato ratio fluorescence intensity expressed relative to baseline) were measured after the experiment in the defined ROI using the Quantify package of LAS X software (3.6.0.20104; Leica-Microsystems)[4–7]. Maximum change in fluorescent intensity was determined by measuring changes in fluorescence intensity in multi-cell regions. Arteriole diameters were measured at baseline and at maximum vasoconstriction in response to vasoconstrictor challenge as reported before[4].

Blood pressure measurement in rodent models. During acute administration of suPAR (50 ng in 50 µL 0.9% saline) in anaesthetised animals, BP was measured using an analog single-channel transducer, signal conditioner model BP-1 (World Precision Instruments) via the carotid artery as described before[5,8,9].

Serum suPAR concentrations in rodent models. To assess circulating suPAR levels relative to injected doses, recombinant mouse suPAR protein (50 ng; UPR-M52H3, ACROBiosystems, Newark, DE) was administered via retro-orbital intravenous injection in wild-type C57BL/6j (RRID:IMSR_JAX:000664) mice (n=9). Serum and urine suPAR levels were measured at baseline, 6-, 12-, and 24-hours post-injection using a mouse uPAR ELISA kit (R&D Systems) (Fig. S5). In uPAR knockout mice (n=7), suPAR was undetectable in both serum and urine under identical conditions, confirming assay specificity. The absence of detectable levels may also reflect rapid clearance. Notably, when we tested different batches of the recombinant suPAR protein using the ELISA assay, the detected concentration was approximately one-fifth of the labelled amount, suggesting potential differences in immunoreactivity between the recombinant protein and the assay standard.

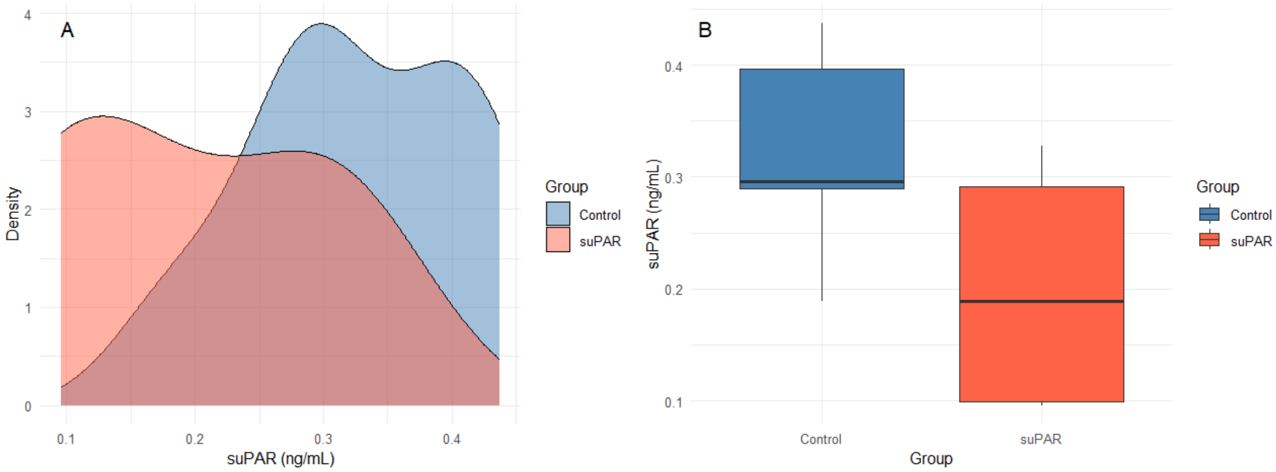

Fig. S1. Baseline suPAR concentration in the porcine model. (*A*) Density plot showing the distribution of baseline suPAR concentrations in the control and intervention (suPAR) groups prior to any treatment. (*B*) Boxplot displaying baseline suPAR concentrations in the two groups, with the median represented by the horizontal line within the box, 25th–75th percentile shown by the box itself, and the minimum and maximum values indicated by the whiskers. Both plots demonstrate comparable baseline suPAR levels between the groups.

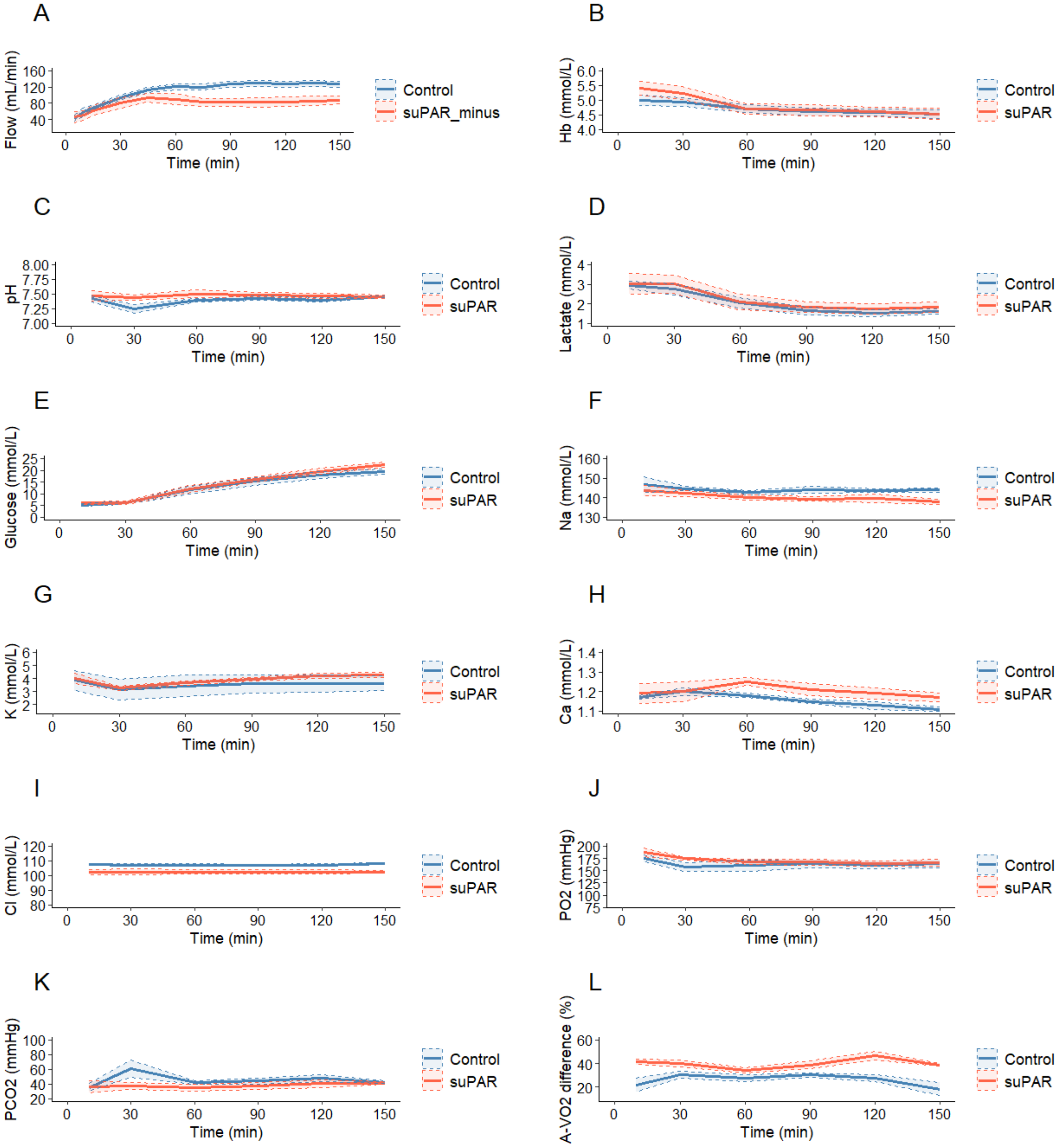

Fig. S2. Physiological and biochemical parameters during *ex vivo* perfusion. (*A*) Flow rate (ml/min) over time comparing control and suPAR groups, (*B*) Haemoglobin (Hb) levels, (*C*) pH values, (*D*) Lactate concentrations, (*E*) Glucose levels, (*F*) Sodium (Na^+) concentrations, (*G*) Potassium (K^+) levels, (*H*) Calcium (Ca^{2+}) concentrations, (*I*) Chloride (Cl^-) levels, (*J*) Partial pressure of arterial oxygen (PaO_2), (*K*) Partial pressure of arterial carbon dioxide ($PaCO_2$), and (*L*) Arteriovenous oxygen saturation (A-VO_2) difference between control and suPAR groups. AV-O_2 differences were deemed unreliable for assessing oxygen extraction due to the open perfusion system, blood loss from suboptimal venous cannulation and biopsy sampling (with exposure to atmospheric gas exchange in the PVC organ carrier), and reinfusion of this blood into the circuit. Data points represent mean ± SEM over 150 minutes of perfusion.

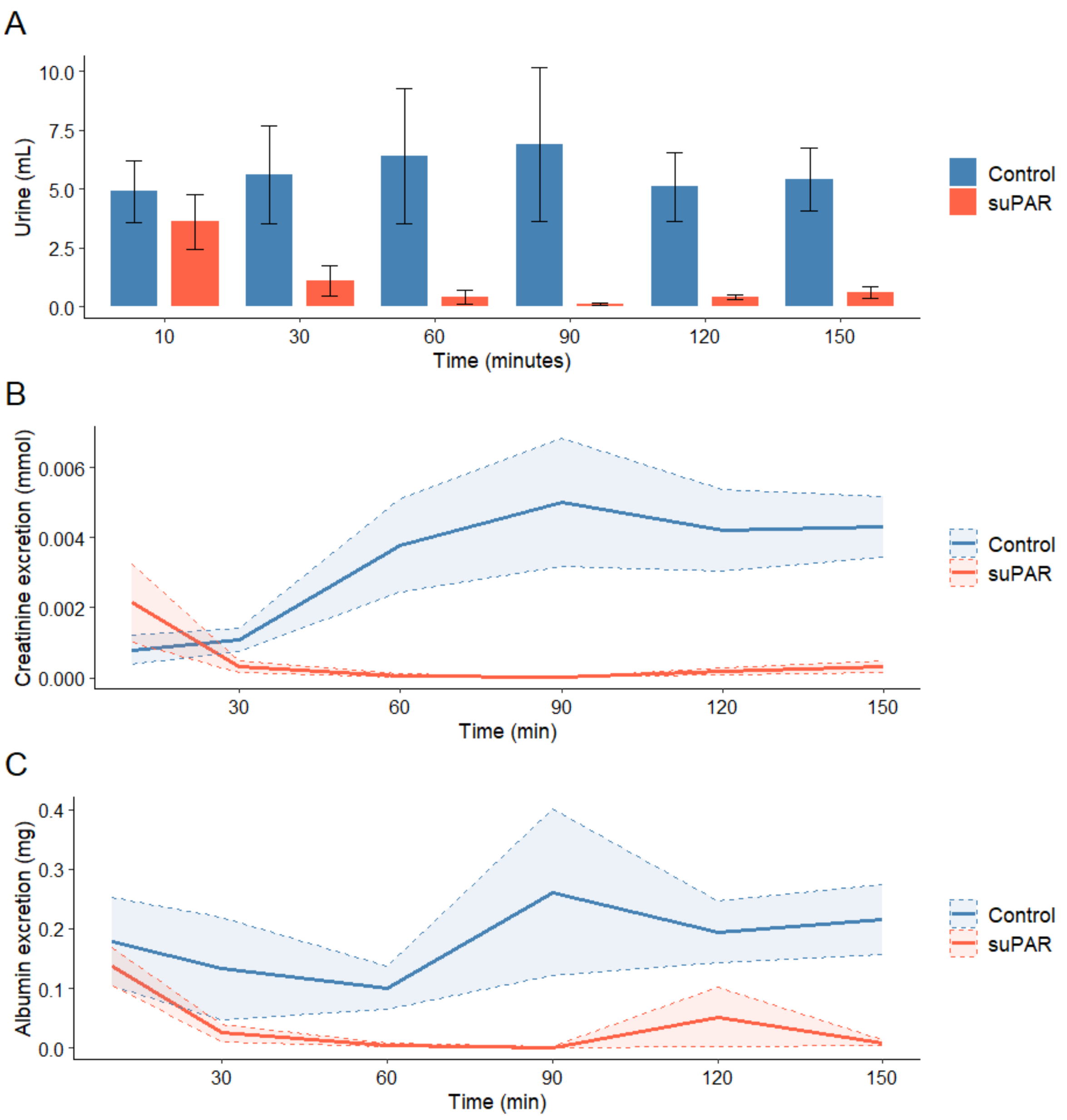

Fig. S3. Urinary parameters during *ex vivo* perfusion. (*A*) Urine output (mL) comparing control and suPAR groups throughout *ex vivo* perfusion, presented as mean ± SEM. (*B*) Urine creatinine excretion and (*C*) urine albumin excretion measured by absorption photometric analysis (Cobas 8000, Roche Diagnostics). Note that six samples could not be analysed due to insufficient urine volume. Data are expressed as mean ± SEM.

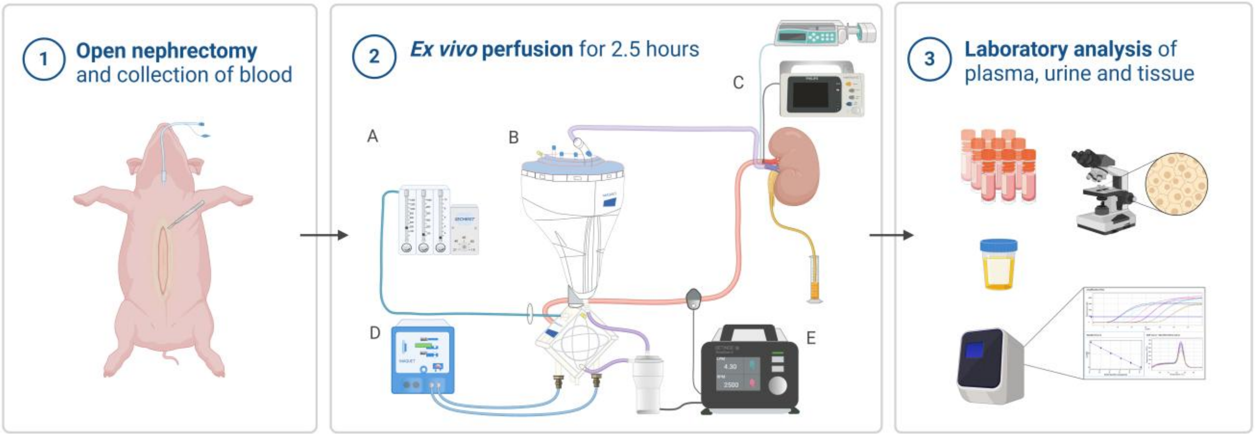

Fig. S4. Schematic presentation of the *ex vivo* perfusion model. (*A*) gas mixer with adjustment of CO_2, O_2, and air, (*B*) hard-shell reservoir with neonatal oxygenator, (*C*) invasive pressure module and infusion line with vasodilator, (*D*) heater-cooler unit, (*E*) extracorporeal life support system with centrifugal pump and ultrasonic flow probe (created with Biorender.com).

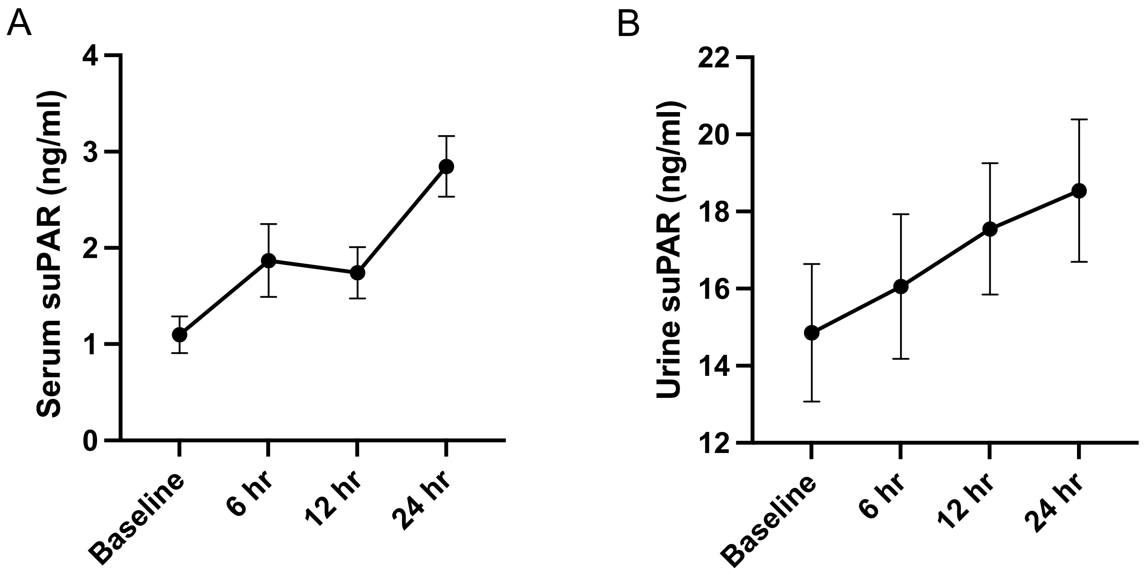

Fig. S5. suPAR levels in wild-type C57BL/6j mice after intravenous administration of recombinant mouse suPAR protein. Mice (n=9, M=5, F=4) received 50 ng recombinant suPAR via retro-orbital injection. (*A*) Serum suPAR levels measured at baseline, 6-, 12-, and 24-hours post-injection, showing a progressive increase over time. (*B*) Urine suPAR concentrations measured at the same time points, demonstrating a gradual rise from baseline to 24 hours. Data are presented as mean ± SEM.

Table S1. Kidney outcomes in cardiac surgery patients after propensity score matching

	Propensity matched populations			
Postoperative outcomes	**High suPAR** n = 146	**Low suPAR** n = 292	*P* value	Adj. *P* value
Acute kidney injury	81 (56%)	95 (33%)	<0.001	<0.001
- *Stage I*	61 (75%)	77 (81%)		
- *Stage 2*	7 (9%)	12 (13%)		
- *Stage 3*	13 (16%)	6 (6%)		
Peak postoperative serum creatinine	131 (109–192)	101 (85–126)	<0.001	<0.001
Absolute increase in serum creatinine	33 (16–62)	19 (7–33)	<0.001	<0.001
Kidney replacement therapy	12 (8.2%)	7 (2.4%)	0.010	0.020

Comparison of postoperative outcomes between patients with high (>4 ng/mL) and low (≤4 ng/mL) suPAR levels after propensity score matching. Data are presented as n (%) for categorical variables and median (25th–75th percentiles) for continuous variables. *P* values were calculated using chi-square test for categorical variables and Mann-Whitney U test for continuous variables. Acute kidney injury was defined and staged according to KDIGO criteria. Adj. *P* values are corrected for multiple testing using the false discovery rate (Benjamini-Hochberg) method[10].

Table S2. Perioperative laboratory values before *ex vivo* perfusion

Variable	**Control group** (n=7)		**suPAR group** (n=5)		*P* value	Adj. *P* value
pH	7.4	(7.4–7.5)	7.4	(7.4–7.4)	0.68	0.79
$PaCO_2$ (mmHg)	45.1	(41.6–49.1)	47.1	(46.7–52.5)	0.37	0.55
PaO_2 (mmHg)	220.0	(185.5–275.0)	176.0	(148.0–183.0)	0.12	0.24
tHb (mmol/L)	6.1	(5.8–6.3)	5.9	(5.6–6.5)	0.62	0.77
SaO_2 (%)	100.2	(99.5–100.4)	100.0	(99.4–100.0)	0.19	0.34
K^+ (mmol/L)	4.1	(3.8–4.8)	4.3	(3.8–4.4)	0.87	0.90
Na^+ (mmol/L)	142.0	(141.5–142.5)	139.0	(137.0–139.0)	0.29	0.47
Ca^{2+} (mmol/L)	1.3	(1.3–1.4)	1.3	(1.3–1.4)	0.68	0.79
Cl^- (mmol/L)	101.0	(99.5–102.5)	98.0	(95.0–99.0)	0.07	0.16
Glucose (mmol/L)	7.1	(6.2–7.6)	8.1	(7.8–8.4)	0.37	0.55
Lactate (mmol/L)	2.5	(2.2–2.7)	1.8	(1.7–3.1)	0.46	0.67

Results are presented as median (25th–75th percentile). Hypothesis test was performed using Wilcoxon-Mann-Whitney Test. Arterial blood gases were sampled shortly after the placement of the sheath in the abdominal aorta and before the collection of whole blood for the *ex vivo* circuit. Adj. *P* values are corrected for multiple testing using the false discovery rate (Benjamini-Hochberg) method[10]. Abbreviations: $PaCO_2$; partial pressure of arterial carbon dioxide, PaO_2; partial pressure of arterial oxygen, tHb; total haemoglobin, SaO_2; arterial oxygen saturation, K^+; potassium, Na^+; sodium, $Ca^{2+;}$ calcium, Cl^-; chloride.

Table S3. Laboratory values during *ex vivo* perfusion

Variable	**Control group** (n=7)		**suPAR group** (n=5)		*P* value	Adj. *P* value
pH	7.4	(7.4–7.5)	7.5	(7.4–7.5)	0.25	0.43
$PaCO_2$ (mmHg)	42.0	(38.6–48.1)	39.9	(36.6–44.3)	0.54	0.69
PaO_2 (mmHg)	165.5	(152.2–175.8)	173.0	(164.0–178.0)	0.18	0.34
tHb (mmol/L)	4.7	(4.5–4.9)	4.9	(4.5–5.1)	0.014	0.039
SaO_2 (%)	103.9	(103.3–104.2)	104.3	(100.0–104.5)	0.96	0.96
AV-SO_2 difference (%)	27.6	(22.8–32.3)	38.9	(35.2–43.7)	0.40	0.59
K^+ (mmol/L)	2.8	(1.9–4.5)	3.5	(3.2–3.9)	0.22	0.39
Na^+ (mmol/L)	143.5	(140.2–145.0)	138.0	(136.0–139.0)	0.056	0.13
Ca^{2+} (mmol/L)	1.2	(1.1–1.2)	1.2	(1.2–1.2)	0.11	0.22
Cl^- (mmol/L)	108	(107–108)	102	(100–106)	0.96	0.96
Glucose (mmol/L)	12.9	(6.6–18.8)	14.6	(6.6–19.7)	0.19	0.34
Lactate (mmol/L)	1.9	(1.6–2.7)	2.1	(1.6–2.7)	0.85	0.90
P-creatinine (µmol/L)	20.3	(18.0–21.1)	18.9	(16.9–20.4)	0.085	0.19
P-albumin (g/L)	40.5	(30.0–59.0)	54.0	(41.5–68.0)	0.022	0.058

Median values (25th–75th percentiles) for the two groups (six samples per animal) during *ex vivo* kidney perfusion. The accompanying *P* values reflect the significance of the interaction term in a linear mixed model analysis, assessing any difference between groups throughout the entire *ex vivo* perfusion duration. Adj. *P* values are corrected for multiple testing using the false discovery rate (Benjamini-Hochberg) method[10]. Abbreviations: $PaCO_2$; partial pressure of arterial carbon dioxide, PaO_2; partial pressure of arterial oxygen, tHb; total haemoglobin, SaO_2; arterial oxygen saturation, K^+; potassium, Na^+; sodium, Ca^{2+}; calcium, Cl^-; chloride, AV-SO_2 difference; difference in arterial and venous oxygen saturation, P-creatinine; plasma creatinine, P-albumin; plasma creatinine. AV-O_2 differences were deemed unreliable for assessing oxygen extraction due to the open perfusion system, blood loss from suboptimal venous cannulation and biopsy sampling (with exposure to atmospheric gas exchange in the PVC organ carrier), and reinfusion of this blood into the circuit**.**

Table S4. Linear mixed model effects estimates on standardised kidney injury and inflammatory biomarkers

Variable	Coefficient	Estimate	Std. Error	df	*P* value	Adj. *P* value
**ELISA results**						
NGAL	(Intercept)	0.020	0.045	21.72	0.65	0.80
	Time	0.0028	0.00040	67.55	<0.001	**<0.001**
	Time:Group	-0.00050	0.00055	58.89	0.37	0.55
**qPCR results**						
NGAL	(Intercept)	-0.21	0.31	24.11	0.51	0.69
	Time	0.0011	0.0029	68.03	0.70	0.81
	Time:Group	0.0064	0.0039	53.58	0.10	0.21
eNOS	(Intercept)	0.36	0.56	19.28	0.53	0.69
	Time	-0.010	0.0046	66.80	0.025	0.064
	Time:Group	0.013	0.0065	63.90	0.048	0.12
TNF-α	(Intercept)	0.088	0.48	18.35	0.86	0.90
	Time	0.0022	0.0038	66.38	0.57	0.72
	Time:Group	-0.0060	0.0054	65.84	0.27	0.45
IL-1β	(Intercept)	0.35	0.49	18.38	0.49	0.69
	Time	0.014	0.0039	66.42	<0.001	**0.002**
	Time:Group	-0.0081	0.0056	65.62	0.15	0.29
IL-6	(Intercept)	-0.31	0.47	22.34	0.52	0.69
	Time	0.011	0.0042	67.71	0.012	**0.034**
	Time:Group	0.0012	0.0058	57.25	0.83	0.90

Effect estimates of the linear mixed model analyses. All qPCR results are normalised to Ribosomal protein L4 (RPL4). Data are further standardised by dividing all individual observations by the starting value at 10 minutes. Adj. *P* values are corrected for multiple testing using the false discovery rate (Benjamini-Hochberg) method[10]. A significant difference between groups over the entire *ex vivo* perfusion duration is indicated by the *P* values of the interaction term (Time:Group). Abbreviation: ELISA; enzyme linked immunosorbent assay, NGAL; Neutrophil gelatinase-associated lipocalin, qPCR; quantitative polymerase chain reaction, eNOS; Endothelial nitric oxide synthase, TNF-α; Tumour necrosis factor-alpha, IL-1β; Interleukin 1 beta and IL-6; Interleukin 6.

Table S5. Linear mixed model effects estimates on hemodynamic parameters

Variable	Coefficient	Estimate	Std. Error	df	*P* value	Adj. *P* value
**Blood flow indexed**	(Intercept)	0.62	0.090	10	<0.001	<0.001
	Time	0.0048	0.00050	133	<0.001	<0.001
	Group	0.0055	0.070	136	0.94	0.96
	Time:Group	-0.0026	0.00077	133	0.0011	0.003
**Perfusion pressure**	(Intercept)	81.53	0.71	9.45	<0.001	<0.001
	Time	0.016	0.0036	132.83	<0.001	<0.001
	Group	-0.22	0.50	135.40	0.66	0.80
	Time:Group	-0.011	0.0055	132.83	0.056	0.13

Effect estimates from the linear mixed model analyses are presented. Blood flow was indexed by dividing the measured blood flow (mL/min) by the kidney weight in grams. Perfusion pressure was measured as side pressure on the arterial cannula using an invasive pressure module. Adj. *P* values are corrected for multiple testing using the false discovery rate (Benjamini-Hochberg) method[10]. A significant difference between groups over the entire *ex vivo* perfusion duration is indicated by the *P* values of the interaction term (Time:Group).

Table S6. Porcine primer sequences

Gene transcript	forward	Reverse
NGAL	CTTCCAGGCTGACCAGTTCC	AGCTCGTAGGTGGTGGTGTA
RPL4	CAAGAGTAACTACAACCTTC	GAACTCTACGATGAATCTTC
eNOS	TCCTTCTCCAACCGCCTAGA	GCTCACTTACGCCCTGATGA
TNF-α	GGCTGCCTTGGTTCAGATGT	CAGGTGGGAGCAACCTACAGTT
IL-1β	GATGACACGCCCACCCTG	CAAATCGCTTCTCCATGTCCC
IL-6	AGACAAAGCCACCACCCCTAA	CTCGTTCTGTGACTGCAGCTTATC

Primer gene sequences used for the study. Abbreviation: NGAL; Neutrophil gelatinase-associated lipocalin, RPL4; Ribosomal protein L4, eNOS; Endothelial nitric oxide synthase, TNF-α; Tumour necrosis factor-alpha, IL-1β; Interleukin 1 beta and IL-6; Interleukin 6.

Movie S1 (separate file). Representative time-lapse recording of GCaMP6f fluorescence intensity (pseudocolour) of a control healthy Sox2-Salsa6f mouse kidney glomerulus (G) with the afferent (AA) and efferent (EA) arterioles, extraglomerular mesangium (EGM, arrow), and macula densa (MD). The time of acute bolus suPAR injection is indicated. Note the most robust calcium increase in cells of the EGM at the base of the macula densa in response to acute suPAR injection (ic. 50 ng in 50 µL 0.9% saline) indicated by the appearance of yellow-red pseudocolour labelling (arrow).

Movie S2 (separate file). *Ex vivo* kidney perfusion system setup. The video demonstrates the complete normothermic *ex vivo* kidney perfusion system including the custom-designed kidney carrier, hard-shell reservoir integrated with a neonatal oxygenator, temperature control via heater-cooler unit, extracorporeal life support system featuring a centrifugal pump and ultrasonic flow probe for precise flow monitoring, invasive pressure measurement module, vasodilator infusion line, and a gas mixer for precise control of CO_2, O_2, and air in the circuit.

Dataset S1 (separate file). Raw data from the clinical and experimental studies.

Supplementary Materials references

1 Hackl MJ, Burford JL, Villanueva K, *et al.* Tracking the fate of glomerular epithelial cells in vivo using serial multiphoton imaging in new mouse models with fluorescent lineage tags. *Nat Med* 2013; **19**: 1661–6.
2 Kang JJ, Toma I, Sipos A, McCulloch F, Peti-Peterdi J. Quantitative imaging of basic functions in renal (patho)physiology. *Am J Physiol Renal Physiol* 2006; **291**: F495-502.
3 Kisler K, Nelson AR, Rege S V, *et al.* Pericyte degeneration leads to neurovascular uncoupling and limits oxygen supply to brain. *Nat Neurosci* 2017; **20**: 406–16.
4 Shroff UN, Gyarmati G, Izuhara A, Deepak S, Peti-Peterdi J. A new view of macula densa cell protein synthesis. *Am J Physiol Renal Physiol* 2021; **321**: F689–704.
5 Becerra Calderon A, Shroff UN, Deepak S, *et al.* Angiotensin II Directly Increases Endothelial Calcium and Nitric Oxide in Kidney and Brain Microvessels In Vivo With Reduced Efficacy in Hypertension. *J Am Heart Assoc* 2024; **13**: e033998.
6 Gyarmati G, Shroff UN, Izuhara A, *et al.* Sparsentan improves glomerular hemodynamics, cell functions, and tissue repair in a mouse model of FSGS. *JCI Insight* 2024; **9**: e177775.
7 Gyarmati G, Shroff UN, Riquier-Brison A, *et al.* Neuronally differentiated macula densa cells regulate tissue remodeling and regeneration in the kidney. *J Clin Invest* 2024; **134**: e174558.
8 Akgür FM, Zibari GB, McDonald JC, Granger DN, Brown MF. Kinetics of P-selectin expression in regional vascular beds after resuscitation of hemorrhagic shock: a clue to the mechanism of multiple system organ failure. *Shock* 2000; **13**: 140–4.
9 Pluznick JL, Protzko RJ, Gevorgyan H, *et al.* Olfactory receptor responding to gut microbiota-derived signals plays a role in renin secretion and blood pressure regulation. *Proc Natl Acad Sci U S A* 2013; **110**: 4410–5.
10 Benjamini Y, Hochberg Y. Controlling the False Discovery Rate: A Practical and Powerful Approach to Multiple Testing. *Journal of the Royal Statistical Society Series B (Methodological)* 1995; **57**: 289–300.
